# Clinical Characteristics of Nasal Fractures: An 11-year Retrospective Study

**DOI:** 10.1055/s-0044-1788314

**Published:** 2025-06-10

**Authors:** Ramyar Farzan, Mohammadjavad Sharifian, Mohammad Tolouei, Siamak Rimaz, Sanaz Masoumi

**Affiliations:** 1 Department of Plastic and Reconstructive Surgery, School of Medicine, Guilan University of Medical Sciences, Rasht, Iran; 2 Guilan University of Medical Sciences, Rasht, Iran; 3 Department of General Surgery, School of Medicine, Guilan University of Medical Sciences, Rasht, Iran; 4 Respiratory Diseases Research Center, Razi Hospital, Guilan University of Medical Sciences, Rasht, Iran; 5 Burn and Regenerative Medicine Research Center, Velayat Burn University Hospital, Guilan University of Medical Sciences, Rasht, Iran

**Keywords:** injuries, maxillofacial injury, fractures, bone, epidemiology

## Abstract

**Introduction** Nasal fractures are one of the most common diseases in the otorhinolaryngology emergency room that leads to significant complications. However, there is still no suitable method to prevent their occurrence, which may result from insufficient studies on their causes and related factors.

**Objectives** To describe the demographic features, pattern, time of consultation, and etiological factors of patients with different types of nasal fractures.

**Methods** We conducted a retrospective study of the records of patients with a diagnosis of nasal fracture treated between 2010 and 2021. The data included demographic characteristics, type of maxillofacial injury and associated lesions, complication rates, treatment modalities, and a description of the surgery.

**Results** We included 599 patients, mostly male subjects (81.6%) injured in a road accident (55.3%), with a mean age of 31.64 ± 14.65 years, and mean length of hospital stay of 2.27 ± 2.21 days. Most accompanying fractures were maxillary (38.5%), multiple (24.6%), and mandibular (23.1%). The mean length of hospital stay was statistically different according to the cause of the fracture ( *p* = 0.036) and the types of treatment performed ( *p* = 0.041).

**Conclusion** In general, trauma patients in the second to fourth decades of life and of the male gender are more prone to nasal fractures. Identifying the factors affecting the incidence of fractures enables the determination of the presentation patterns and the nature of the lesions to be evaluated. In addition, treatment evaluation and an analysis of the complication rate enable a more realistic interpretation of how patients are managed.

## Introduction

Nasal bone fracture is the most common facial bone fracture and one of the most common acute diagnoses managed by Ear, Nose, and Throat (ENT) departments. 1 2 The nose protrudes from the center of the face and the nasal bone is protected only by a thin layer of skin and fatty tissue, making it susceptible to impact; the prominent and delicate structure of the nose causes nasal fractures to occur frequently. 2 3 4 In adult and pediatric patients, the most common causes of nasal fractures differ in different studies and are related to fights, traffic accidents, sports, and falling from a height. 1 5 6 The prevalence of nasal fracture in both adults and children is higher among male individuals and increases significantly in the second and third decades of life. 1 7

In addition, there are significant emotional, functional, and cosmetic repercussions associated with nasal trauma. 8 The nose's appearance is one of the most compelling issues involved in the beauty of the face, and failure to diagnose and adequately treat nasal fractures leads to structural and functional deformities of the nose. 9 However, they are often unrecognized and untreated after an injury. 6 The rate of complications in nasal fracture are estimated to range from 30 to 40%, and they include septal deviation, nasal bone deformity, transient loss of smell, and syncytia. Nasal bone deviation, nasal hump, flat nose, and mild nasal irregularities are different forms of nasal bone deformity. Nasal deformity and septal deviation are the most common complications of nasal bone fracture respectively. 10 The second aspect of nasal bone injuries is legal: the importance lies in the frequency of injuries, diverse approaches to diagnosis, treatment, and findings of injuries used in legal proceedings in court. 6

Numerous papers have been published about nasal bone fractures, 2 but epidemiological studies in this field are insufficient. Epidemiological studies are necessary to determine the needs of each population to improve the quality of life and health. 11 Epidemiological investigation of nasal traumas has provided valuable information about the type of injury, as well as knowledge of the kind of geographical region, socioeconomic status, and social and traffic behaviors – which are effective causes of trauma –, which are of great help in planning and creating solutions, such as enacting laws and implementing initiatives for public health. Moreover, data on factors such as occupation, consumption of alcohol and drugs, use of safety equipment, and types of accidents can help strengthen and accurately enact appropriate preventive laws, but there is a need for more epidemiological studies in this field. The present study aimed to conduct an epidemiological investigation of nasal fractures in patients in Northern Iran for 11 years.

## Methods

We conducted a retrospective study with 599 nasal bone fracture patients in an 11-year period, from January 2011 to December 2021.

**Fig. 1:**
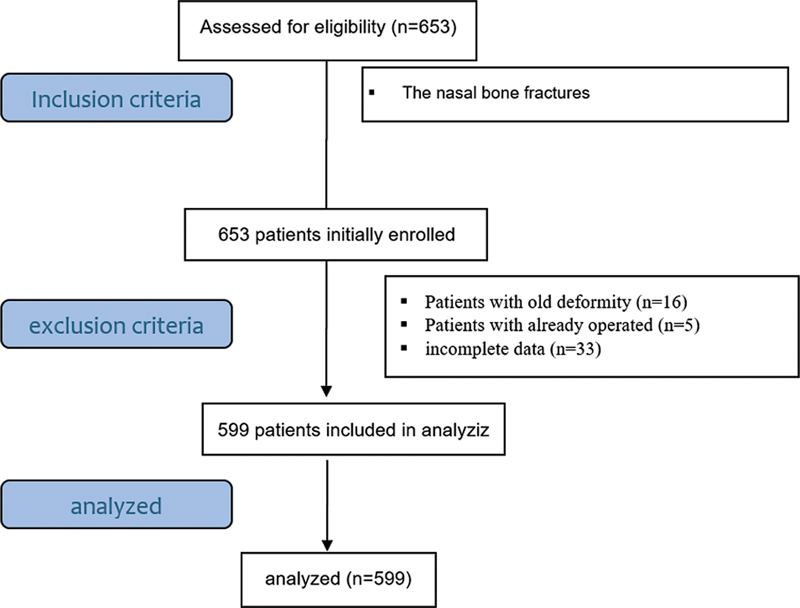
Flowchart of the present study.

The inclusion criteria were as follows: the nasal bone fracture was confirmed by the clinical history, physical exam, and, in some cases, nasal bone radiography. The patients who presented with history of nasal trauma without fracture confirmed on physical examination or radiography were excluded, and all patients with a history of chronic pathology, old deformity, and those who had already been operated on were not included. The sampling method was census. Among the 673 patients initially enrolled, 599 met the inclusion criteria and were considered in the final data analysis ( Fig. 1 ).

The epidemiology and patterns of nasal bone fractures were investigated based on patient medical records. Demographic information was recorded, including name, age, sex, and address. The history of their current problem was obtained regarding symptoms, severity, and duration. They were examined for positive signs, types, and severity. Routine investigations, such as nasal bone radiographs in the anteroposterior (AP), and lateral views were performed. Special studies, such as computed tomography (CT) scans, were performed whenever necessary or whenever there was a medicolegal requirement. The nasal bone fractures were classified as follows: type I – simple without displacement; type II – simple with displacement/without telescoping; and type III – comminuted with telescoping or depression.

### Statistical Analysis

The continuous variables were expressed as mean ± standard deviation values, and the categorical variables, as numbers and percentages. The Fisher exact test was used for dichotomous variations; the Chi-squared test was applied for organized categorical variables. Analysis of variance (ANOVA) was employed to analyze continuous measures. All data were analyzed in a two-tailed manner, and values of *p* < 0.05 were deemed statistically significant for the aims of the present paper. The IBM SPSS Statistics for Windows (IBM Corp., Armonk, NY, United States) software, version 23.0, was used for all analyses.

## Results

**Fig. 2:**
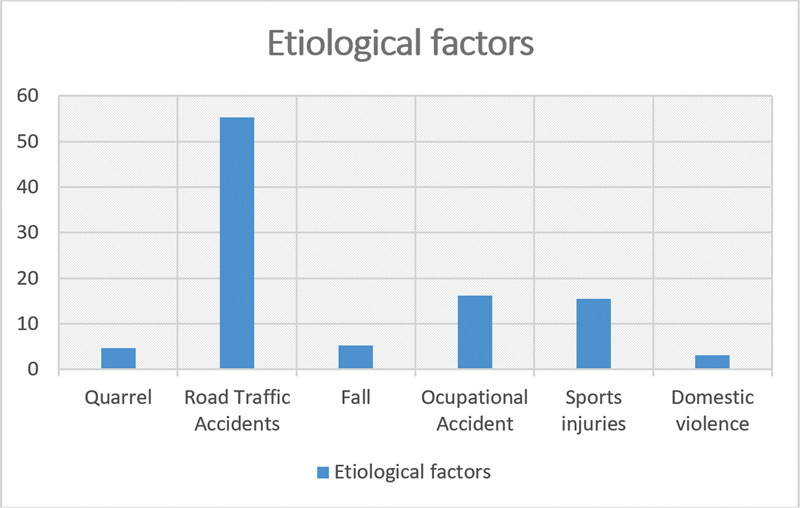
Causes of nasal fractures.

A total of 599 patients were enrolled in the study, and their basic and clinical characteristics are listed in Table 1 . The mean age of the patients was of 31.64 ± 14.65, with a minimum of 1 and a maximum of 83 years. The mean hospital stay was of 2.27 ± 2.21 days, with a minimum of 1 and a maximum of 25 days. In total, 91.2% of the patients did not have other associated fractures, which were only presented by 8.8% of the subjects. Overall, 7.3% of the patients presented 1 fracture, 1%, 2 fractures, and only 0.5%, 3 fractures associated with the nasal fractures ( Table 1 ). The most common causes of the fractures were fights, accidents, falls, and other reasons, such as domestic violence, sports injuries, and occupational injuries ( Fig. 2 ).

**Table 1:** Basic and clinical characteristics of the patients

Parameters	Status	N (%)	Parameters	Status	N (%)
Gender	Male	489 (81/6)	Etiological factors	Quarrel	28 (4/7)
	Female	110 (18/4)		Traffic accidents	331 (55/3)
Age (years)	≤ 10	15 (2/5)		Fall	31 (5/2)
	11–20	128 (21/4)		Others	209 (34/9)
	21–30	194 (32/4)			
	40–31	124 (20/7)			
	50–41	69 (11/5)			
	60–51	40 (6/7)	Associated fractures	Yes	53(8/8)
	≥ 60	29 (4/8)		No	564 (91/2)
Day the fracture occurred	Working day	517 (86/3)			
	Holiday	82 (13/7)			
Length of hospital stay (days)	< 1	81 (13/5)	Types of associated fractures	Skull base fracture	7 (10/8)
	1	186 (31/1)		Maxillary fracture	25 (38/5)
	2–3	214 (35/7)		Mandibular fracture	15 (23/1)
	4–7	102 (17)		Multiple fractures	16 (24/6)
	≥ 7	16 (2/7)		Other fractures	2 (3/1)
Season in which the fracture occurred	Spring	146 (24/4)	Treatment	Closed treatment	355 (59/3)
	Summer	157 (26/2)		Open reduction and fixation	153 (25/5)
	Fall	151 (25/2)		Without fixation	86 (14/4)
	Winter	145 (24/2)		Unknown	5 (0/8)

The results showed a significant statistical relationship involving gender, length of hospital stay, and the types of procedures performed with the causes of nasal fractures. However, there was no significant statistical relationship between age, day of admission to the hospital, accompanying fracture, and the season of referral of patients with nasal fractures with the causes of those fractures ( Table 2 ).

**Table 2:** Demographic characteristics of the patients and mechanism of nasal fracture

Parameters	Status	Quarrel		Traffic accidents		Fall		Others		*p* -value*
		N	%	N	%	N	%	N	%	
Gender	Male	25	3/89	280	6/84	20	5/64	164	5/78	0.015
	Female	3	7/10	51	4/15	11	5/35	45	5/21	
Age (years)	≤ 10	8	6/28	70	1/21	6	4/19	59	2/28	479/0
	11–20	9	1/32	116	35	8	8/25	61	2/29	
	21–30	6	4/21	74	4/22	7	6/22	37	7/17	
	≥ 60	5	9/17	71	5/21	10	3/32	52	9/24	
Day the fracture occurred	Working day	24	7/85	290	6/87	25	6/80	178	2/85	669/0
	Holiday	4	3/14	41	4/12	6	4/19	31	8/14	
Length of hospital stay (days)	< 1	2	1/7	30	1/9	8	8/25	41	6/19	0.016
	1	9	1/32	100	2/30	8	8/25	69	33	
	2–3	11	3/39	125	8/37	9	29	69	33	
	4–7	6	4/21	66	9/19	4	9/12	26	4/12	
	≥ 7	0	0	10	3	2	5/6	4	9/1	
Associated fractures	Yes	3	7/10	33	10	3	7/9	14	7/6	602/0
	No	25	3/89	298	90	28	3/90	195	3/93	
Season in which the fracture occurred	Spring	6	4/21	80	2/24	10	3/32	50	9/23	697/0
	Summer	5	9/17	95	7/28	6	4/19	51	4/24	
	Fall	11	3/39	77	3/23	7	6/22	56	8/26	
	Winter	6	4/21	79	9/23	8	8/25	52	9/24	
Treatment	Closed treatment	18	3/64	510	4/63	27	1/87	100	8/47	0001/0
	Open reduction and fixation	4	3/14	86	26	0	0	63	1/30	
	Without fixation	6	4/21	35	6/10	4	9/12	41	6/19	
	Unknown	0	0	0	0	0	0	5	4/2	

**Note:** *Chi-squared test; Fisher exact test.

Moreover, the presence of an accompanying fracture, the cause of the fracture, and the types of treatment presented a statistically significant relationship with the length of hospital stay. However, the length of stay was not significantly related to age, gender, day of visit to the hospital, and season of visit ( Table 3 ).

**Table 3:** Demographic characteristics of patients and length of hospital stay

Parameters	Status	Mean ± standard deviation	*p* -value*
Gender	Male	99/1 ± 3/2	469/0
	Female	99/2 ± 13/2	
Age (years)	≤ 10	98/1 ± 18/2	189/0
	11–20	87/1 ± 05/2	
	21–30	1/2 ± 36/2	
	≥ 60	87/2 ± 57/2	
Day the fracture occurred	Working day	05/2 ± 2/2	066/0
	Holiday	03/3 ± 68/2	
Length of hospital stay (days)	< 1	02/2 ± 46/2	026/0
	1	03/2 ± 44/2	
	2–3	67/4 ± 71/2	
	4–7	9/1 ± 9/1	
	≥ 7	66/2 ± 09/4	
Associated fractures	Yes	08/2 ± 09/2	0001/0
	No	66/1 ± 84/1	
Season in which the fracture occurred	Winter	03/2 ± 08/2	062/0
	Spring	67/2 ± 48/2	
	Summer	3/2 ± 37/2	
	Fall	01/2 ± 35/2	
Treatment			041/0
	Closed treatment	86/1 ± 52/2	
	Open reduction and fixation	24/3 ± 65/2	
	Without fixation	44/0 ± 2/1	

**Note:** *Analysis of variance.

## Discussion

Nasal bone fracture is a common problem in the ENT practice. Few works address nasal trauma separately; such a topic is generally approached along with general facial trauma. As the most prominent position organ in the face, it is the most affected location in facial trauma, and it is possible to compare the etiology of nasal trauma separately from that of facial trauma.

The present study assessed 599 patients with an average age of 31.64 years. In terms of age distribution, most of the patients were in the third, second, and fourth decades of life, respectively, which is consistent with almost all other studies on nasal fractures. 10 12 13 14 In justifying these findings, it should be mentioned that the second and third decades of life are the most active period, when people are often trying to make a living and performing activities outside the home, so they are more exposed to high-risk conditions, such as traffic accidents. 15 16 We have observed that the results of most of the studies on the pattern of maxillofacial fractures align with those of our research. In a retrospective study by Shirinback et al. 16 (2018), the most common age group was between 21 and 31 years. Also, in a cross-sectional survey by Kazemiyan et al. 17 (2014), the patients were aged between 21 and 30 years. Jalali et al. 18 (2015) reported that most nasal fracture patients were aged between 21 and 30 years. 18 In a study on the incidence and patterns of maxillofacial trauma conducted by Manodh et al., 19 in 2016, in India, maxillofacial injuries were more common in the third decade of life. However, Zahedi et al. 20 (2017) found that the mean age of patients with nasal fractures was of 68.26 ± 47.14, which is inconsistent with the results of the current study and other similar studies.

The results of the present study show that most patients were male (86.6%), which aligns with other studies on nasal fractures. 6 21 22 23 In a 2021 systematic review, Jaber et al. 24 showed that men outnumbered women by a ratio of 4.5 to 1. In the study by Boffano et al. 25 (2014), the ratio of men to women was of 2 to 1. In 2014, Arangio et al. 26 conducted a retrospective study in Lazio, Italy, investigating maxillofacial fractures in patients treated from 2011 to 2012, and male patients were more involved in all cases, with a ratio of 4 to 1. Manodh et al. 19 showed that maxillofacial injuries are more common in men. In the study by Shirinback et al., 16 82% of the patients were male, and 18%, female. 16 This can be caused by the physical differences and mental moods of men compared with women. In this way, men generally display riskier behaviors, and as the family's primary breadwinner, they often work outside the home. Therefore, the possibility of violent and aggressive behaviors occurring during conflicts, incidents, and accidents is more likely among men. 15 18 On the other hand, according to the results of the present study, among the different mechanisms of nose damage in the case of quarrels, the contribution of men is higher than that of the rest of the mechanisms, and this difference is statistically significant. Moreover, no significant difference was found regarding the length of hospital stay between men and women.

According to the results of the present study, the leading cause of nasal fractures was accidents (55.3%), which is in line with many similar studies regarding these fractures. 12 16 22 The etiology of maxillofacial injuries changes from center to center, 27 and, in American, African, and Asian studies, road traffic crashes were the predominant cause, 25 but in some studies, including a systematic review, 5 reasons such as violence and sports injuries have been mentioned as the leading cause of nasal fracture. 1 17 21 Jaber et al. 24 (2021) mentioned road traffic accidents followed by falls as the most common causes of fractures. In India, traffic accidents, 19 were among the most common causes of nasal fractures and maxillofacial injuries and, in Iran, falling from a height and road accidents. 17 18 20 In European studies, the etiology varies, with assaults and car crashes being the most important factors. 25 They usually have the lowest amount. 28 In general, the most effective solution to reduce maxillofacial fractures following traffic accidents is to make the vehicles and roads safe according to global standards and to improve people's culture in the field of using safety devices while driving. This statement is backed by the fact that most previous studies that reported accidents as the main cause of nasal and maxillofacial fractures were published before those that reported conflict and violence as the leading cause of nasal injuries, which can indicate the positive effect of standardization throughout time of aspects pertaining to roads, vehicle safety, and public education, such as campaigns to increase seat belt use.

The associated fractures among the patients examined in the present study had a frequency of 8.8%, which was lower than the rates found in almost all similar studies. 13 29 30 The presence of a concomitant fracture indicates that the mechanism of the fracture is more severe. It has been shown that accidents or falling from a high height are more often associated with simultaneous fractures of other facial bones. 31 However, the present study did not show a significant difference between the percentage of concomitant fractures and the mechanisms. Compared with other studies, the lower average length of hospital stay, and the frequency of concomitant fractures indicate that the patients with nasal fractures referred to Velayat Hospital generally presented milder injuries than those of the patients in other studies. However, as in similar studies, the most common associated fracture was maxillary fracture, followed by multiple fractures and mandibular fracture. The results of the current study show that most of the patients underwent closed reduction treatment, which is in line with the study of Bakardjiev and Pechalova, 32 who reported that in most of patients, the fractures were treated by closed reduction.

The current study showed that male patients aged 20 to 40 years are more exposed to facial fractures caused by vehicle accidents, so we suggest that this population, as a target group, should be trained in driving safety tips.

## Conclusion

Overall, the evaluation of the epidemiological features of nasal fractures showed that trauma patients in the second to fourth decades of life and of the male gender are more prone to nasal fractures. It seems that the results of epidemiological studies vary according to the geographical area under investigation, as well as to the population examined, which can be related to the fact that populations differ in terms of social, economic, cultural, and environmental issues, traffic laws, not to mention individual differences regarding physique, spirituality, and psychological state.
